# Cytoplasmic p21 is a potential predictor for cisplatin sensitivity in ovarian cancer

**DOI:** 10.1186/1471-2407-11-399

**Published:** 2011-09-21

**Authors:** Xi Xia, Quanfu Ma, Xiao Li, Teng Ji, Pingbo Chen, Hongbin Xu, Kezhen Li, Yong Fang, Danhui Weng, Yanjie Weng, Shujie Liao, Zhiqiang Han, Ronghua Liu, Tao Zhu, Shixuan Wang, Gang Xu, Li Meng, Jianfeng Zhou, Ding Ma

**Affiliations:** 1 Department of Gynecology & Obstetrics, Nanshan People's Hospital, Guangdong Medical College, Shenzhen, Guangdong, 518052, China; 2 Cancer Biology Research Center, Tongji Hospital, Tongji Medical College, Huazhong University of Science and Technology, Wuhan, Hubei, 430030, China; 3 Department of Pediatrics, Maternal and Child Health Hospital of Shenzhen, Southern Medical University, Shenzhen, Guangdong, 518038, China; 4 Department of Gynecology & Obstetrics, People's Hospital of Shenzhen, Shenzhen, Guangdong, 518020, China

## Abstract

**Background:**

P21^(WAF1/Cip1) ^binds to cyclin-dependent kinase complexes and inhibits their activities. It was originally described as an inhibitor of cancer cell proliferation. However, many recent studies have shown that p21 promotes tumor progression when accumulated in the cell cytoplasm. So far, little is known about the correlation between cytoplasmic p21 and drug resistance. This study was aimed to investigate the role of p21 in the cisplatin resistance of ovarian cancer.

**Methods:**

RT-PCR, western blot and immunofluorescence were used to detect p21 expression and location in cisplatin-resistant ovarian cancer cell line C13* and its parental line OV2008. Regulation of cytoplasmic p21 was performed through transfection of p21 siRNA, Akt2 shRNA and Akt2 constitutively active vector in the two cell lines; their effects on cisplatin-induced apoptosis were evaluated by flow cytometry. Tumor tissue sections of clinical samples were analyzed by immunohistochemistry.

**Results:**

p21 predominantly localizes to the cytoplasm in C13* compared to OV2008. Persistent exposure to low dose cisplatin in OV2008 leads to p21 translocation from nuclear to cytoplasm, while it had not impact on p21 localization in C13*. Knockdown of cytoplasmic p21 by p21 siRNA transfection in C13* notably increased cisplatin-induced apoptosis through activation of caspase 3. Inhibition of p21 translocation into the cytoplasm by transfection of Akt2 shRNA into C13* cells significantly increased cisplatin-induced apoptosis, while induction of p21 translocation into the cytoplasm by transfection of constitutively active Akt2 in OV2008 enhanced the resistance to cisplatin. Immunohistochemical analysis of clinical ovarian tumor tissues demonstrated that cytoplasmic p21 was negatively correlated with the response to cisplatin based treatment.

**Conclusions:**

Cytoplasmic p21 is a novel biomarker of cisplatin resistance and it may represent a potential therapeutic target for ovarian tumors that are refractory to conventional treatment.

## Background

Ovarian cancer is the sixth most common cancer among women and it leads to the highest mortality per year than any other cancers of the female reproductive system [1,2]. Cisplatin derivatives are first-line chemotherapeutic agents used for treatment of ovarian cancer. However, chemoresistance remains a major hurdle to successful therapy and results in low five-year survival rates [2,3]. Previous studies have suggested that resistance may be due to reduced drug accumulation [4], increased levels of glutathione and metallothionein [5] and enhanced DNA repair [6]. At present, it is widely accepted that the apoptotic response of cancer cells to chemotherapeutic drugs is the determining force for sensitivity to chemotherapy [7,8]. Many molecules, including XIAP [3], MKP3 [9], PI3K, Akt2 [10,11], PTEN [12], P-glycoprotein [13] and MDR [14], have been reported to be involved in the regulation of apoptosis and in the complicated signaling network that determines the fate of cancer cells, i.e., either "death" or "survival." Though much progress has been made, our recent studies have made efforts to predict the response of cancer cells to chemotherapeutic agents before treatment and to identify the possible alterations that mediate resistance.

P21 was the first identified inhibitor of cyclin/cyclin-dependent kinase (CDK) complexes [15]. Previous studies demonstrated that p21 could act as a "tumor suppressor" by binding to cellular CDK and proliferating cell nuclear antigen (PCNA), thereby inhibiting their function and leading to cell cycle arrest, leading to blockade of DNA synthesis and inhibition of cell proliferation [15,16]. Numerous studies that analyzed the expression of p21 in different types of human cancers have revealed that loss of p21 correlates with carcinogenesis and a poor prognosis in small-cell lung, colorectal, cervical and head and neck cancers [17-20]. In contrast, other findings have found that increased p21 expression was associated with tumor progression in ovarian, cervical, breast and esophageal squamous cell carcinomas [21-26]. This discrepancy could be due to the status of p21 itself and/or to differences in the histological types of cancers that have been analyzed. Besson et al. [27] suggested that control of the subcellular localization of p21 could represent an important regulatory switch from a nuclear tumor suppressor to a cytoplasmic oncogene. Once phosphorylated by Akt, p21 is induced to emigrate from the nucleus to the cytoplasm, protecting cells from apoptosis [28-31]. Additionally, clinical immunohistochemical analysis have proven that cytoplasmic p21 is a novel predictor of poor prognosis in breast cancer [32,33]. Although the role of p21 in the development of various types of human cancers has garnered much attention, little is known about its involvement in drug resistance.

Given that chemoresistance is a biological trait of tumor malignancy and has a direct influence on a patient's prognosis, this study was designed to explore whether cytoplasmic p21 correlated with cisplatin resistance in ovarian tumors. In addition, we sought to determine whether interfering with cytoplasmic p21 could enhance the susceptibility of cancer cells to cisplatin. Here, we report that in the cisplatin-resistant cell line C13*, p21 is predominantly localized to the cytoplasm, while in the cisplatin-sensitive cell line OV2008, p21 is mainly restricted to the nucleus. Additionally, the exposure to low-dose cisplatin in OV2008 induced translocation of most p21 protein from nuclear to cytoplasm. Knockdown of cytoplasmic p21 by transfection of p21 siRNA into C13* cells notably enhanced their sensitivity to cisplatin. Inhibition of p21 translocation into the cytoplasm by Akt2 shRNA transfection in C13* cells significantly enhanced their sensitivity as well. Conversely, the accumulation of p21 in the cytoplasm by transfection of active Akt2 in OV2008 conferred resistance to cisplatin in OV2008 cells. Further analysis of clinical ovarian tumor samples by immunohistochemistry revealed that p21 was predominantly localized in the nucleus in the drug sensitive group. In contrast, p21 is mainly localized in the cytoplasm in the drug resistant group.

## Methods

### Cell lines and cell culture

The cisplatin-resistant ovarian cancer cell line C13* and its parental variant OV2008 were provided by Doctor Benjamin K. Tsang (Department of Obstetrics, Gynecology and Cellular and Molecular Medicine, University of Ottawa, Canada). Cells were maintained in RPMI-1640 supplemented with 2 mM L-glutamine, 100 U/ml penicillin, 100 mg/ml streptomycin and 10% fetal bovine serum (FBS) at 37°C in a humidified atmosphere containing 5% CO_2_.

### Clinical samples

From 2004 through 2007, 40 patients who were diagnosed with ovarian carcinoma and received standard cisplatin-based intravenous chemotherapy after surgery at Tongji Hospital, Tongji Medical College, Huazhong University of Science and Technology were included in the study after obtainment of oral and written informed consents. Paraffin-embedded tumor tissue sections of each patient were prepared by the Department of Clinical Pathology. Information on histopathologic diagnosis was extracted from medical records and reviewed by a specialist in gynecologic pathology. The patients were selected and divided into treatment response and treatment non-response groups according to the CA125 criteria proposed by the Gynecological Cancer Intergroup (GCIC) [34]. Briefly, treatment response patients were defined as having at least a 50% reduction in CA125 levels, when compared to pretreatment samples, and this reduction must have been maintained for at least 28 days; the intervening value of CA125 must have been less than or equal to the previous value. Patients demonstrated any clinical evidence of progression, such as increased lump size and ascites, were excluded from the treatment response group. The remaining patients were classified as treatment non-response group. This study was reviewed and approved by the ethics committee of the medical faculty at the Tongji Hospital, Tongji Medical College, Huazhong University of Science & Technology.

### Antibodies and reagents

Antibodies against Akt, Ser473-phosphorylated Akt and p21 were purchased from Cell Signaling Technology, Inc. (Beverly, MA, USA). β-actin antibody and secondary goat anti-rabbit and goat anti-mouse alkaline phosphatase antibodies were purchased from Santa Cruz Biotechnology (Santa Cruz, CA, USA). Anti-caspase 3 antibody was purchased from Biolegend Co. (San Diego, CA, USA). RPMI-1640, FBS, Lipofectamine 2000 and Trizol reagent were purchased from Invitrogen Co. (Carlsbad, CA, USA). Cisplatin, MTT, DMSO, G418 and DAPI dye were obtained from Sigma Chemical Co. (St. Louis, MO, USA). The NE-PER cytoplasmic protein extraction kit was purchased from Pierce Co. (Rockford, IL, USA). The annexin-V/PI apoptosis detection kit was purchased from Promoter Biotech Ltd. (Wuhan, Hubei, China).

### Construction of plasmids

A constitutively active Akt expression vector (AAkt2) and short hairpin RNA targeting Akt2 (Akt2Sh) were described previously [11,35]. The sequences of the p21 RNA interfering fragment (p21si) were as follows: sense, 5'- CUU CGA CUU UGU CAC CGA G -3'; anti-sense, 5'- C UCG GUG ACA AAG UCG AAG -3' [36]. The sequences of the mismatched fragment (p21sm) were: sense, 5'- CUC GAC UUC GUA CCC GAG -3': anti-sense, 5'- CUC GGG UAC GAA GUC GAG -3'.

### Transient transfection for RNAi targeting

For RNAi targeting, C13* cells cultured in 6-well plates were transfected with indicated plasmids using Lipofectamine 2000. After 6 hours of incubation, the transfection solution was removed, and was replaced with fresh complete growth medium. 48 hours post-transfection the cells were assayed for the expression of p21 and treated with cisplatin for further experiment.

### Establishment of stable-expression cell lines of AAkt2 in OV2008 cells and Akt2sh in C13* cells

OV2008 cancer cells were stably transfected with AAkt2 vector, C13* cancer cells were stably transfected with Akt2sh, using Lipofectamine 2000. Their corresponding empty vectors, i.e., pcDNA3.1 and pEGFPC1, were transfected as negative control. The cells were selected with G418. The concentration of G418 for selection and maintenance was 600 μg/μl. After three weeks the G418-resistant cell pools were established and seeded into 100 mm dishes for further propagation.

### Real-time PCR

Total RNA was isolated from each group of cells using Trizol Regent, according to the manufacturer's instruction. Real-time PCR amplifications were carried out using DNase I (Promega)-treated total RNA. Reactions were performed in a Stratagene MX3000P system using the Real-time PCR Master Mix (TOYOBO, Japan). P21 primer sequences were as follows: sense, 5'-CCT CTT CGG CCC GGT GGA C-3': anti-sense, 5'-CCG TTT TCG ACC CTG AGA G-3'. GAPDH primer sequences were as follows: sense, 5'-ACG GAT TTG GTC GTA TTG GG-3'; anti-sense, 5'-TGA TTT TGG AGG GAT CTC GC-3'.

### Western blot analysis

Total proteins were extracted by lysing cells in buffer containing 50 mM Tris pH 7.4, 150 mM NaCl, 0.5% NP-40, 50 mM NaF, 1 mM Na_3_VO_4_, 1 mM phenylmethylsulfonyl fluoride, 25 mg/ml leupeptin and 25 mg/ml aprotinin. The lysates were cleared by centrifugation, and the supernatants were collected. Cytoplasmic proteins were extracted using the N-PER cytoplasmic protein extraction kit according to manufacturer's instructions. Equal amounts of protein lysate were used for western blot analyses. Specific signals were visualized with NBT/BCIP.

### Cell viability assay using MTT

Cells were seeded into 96-well plates, treated with different concentrations of cisplatin for 24 hours and then assessed using 3-[4, 5-dimethylthiazol-2-yl]-2, 5-diphenyltetrazolium bromide. Cell viability was determined by measuring the optical absorbance of cells at 570 nm wavelength and normalizing the values to the corresponding controls.

### Analysis of apoptosis by flow cytometry

Cells were harvested, washed with PBS and stained with the annexin-V/PI apoptosis kit according to manufacturer's instructions. Analysis of apoptotic cells was performed using a FACScan flow cytometer, and the data were analyzed using cell fit software.

### Immunofluorescence

Cells were trypsinized and plated onto chamber slides for 12 hours. After fixation in acetone, the slides were blocked with BSA, incubated with p21 antibody and rhodamine-conjugated secondary antibody and counterstained with DAPI. Slides were then observed on a confocal laser-scanning microscope (Olympus IX81, Japan). P21 staining was categorized as negative, nuclear or cytoplasmic according to a standard described before [32]. Briefly, negative was defined as undetectable cytoplasmic or nuclear staining. Nuclear p21 was defined as the fraction of tumor cells with positive nuclear staining greater than or equal to that of positive cytoplasmic staining. Cytoplasmic p21 was defined as the fraction of cytoplasmic staining greater than that of nuclear staining. Five randomized fields were counted in order to calculate the percentage of cells that stained with anti-p21 antibody.

### Immunohistochemistry

Paraffin-embedded tissue sections were deparaffinized. After antigen retrieval, slides were incubated with 3% H_2_O_2 _to inhibit endogenous peroxidase. Slides were then blocked with 5% normal serum and incubated with anti-p21 antibody. For qualitative identification of specific antibody staining, the DAKO Envision+ system was used, according to manufacturer's instructions, as described below. After detection with chromogen diaminobenzidine, sections were counterstained with hematoxylin and mounted. For the negative control, all incubation steps were identical except that PBS was used instead of primary antibody. The immunoreactivity of p21 was categorized as negative, nuclear or cytoplasmic according to the standard decribed before [32].

### Image analysis

Western blot images were captured and quantified using the ChemiImager 5500 system from the Alpha Innotech Corporation (San Leandro, CA, USA).

### Statistical analysis

All experiments were repeated three times. The relationship between patients' clinical characteristics and results of p21 immunohistochemistry was assessed using the chi-squared (χ^2^) test. Results expressed as mean ± SD were analyzed using the Student t test. Differences were considered significant when p < 0.05. Data were analyzed using SPSS software version 13.0 (SPSS Inc., Chicago, IL).

## Results

### Cisplatin-induced cytotoxicity and apoptosis in resistant and sensitive cell lines

**Figure 1:**
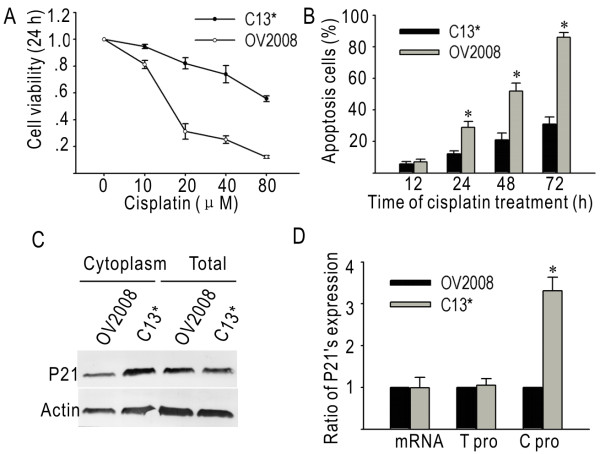
Cisplatin-induced cytotoxicity and apoptosis, and expression of p21 in C13* and OV2008 cells. (A) Dose-response curves for C13* and OV2008 cells following treatment with cisplatin. (B) Cisplatin-induced apoptosis at different time points (20 μM). (C) Comparison of p21 protein levels between paired cell lines. (D) The p21 expression ratio of C13* to OV2008 cells. (*, p < 0.05).

The MTT assay was used to examine the effects of cisplatin on the proliferation of C13* and OV2008 cells. The cells were exposed to different doses of cisplatin for 24 hours. As shown in Figure 1A, cisplatin decreased the viability of both C13* and OV2008 cells in a dose-dependent manner. However, OV2008 cells were more sensitive to cisplatin, exhibiting an approximately 6-fold greater decrease in viability than C13* cells, as evaluated by the IC_50 _value (p < 0.05). The difference between cisplatin-induced viability inhibition of the two cell lines peaked at 51.9% with 20 μM cisplatin (p < 0.05). By flow cytometry, we observed that cisplatin treatment increased apoptosis of OV2008 and C13* cells in a time-dependent manner (Figure 1B). After 24 hours of exposure to cisplatin, there was at least a 2-fold increase of apoptotic OV2008 cells when compared to C13* cells.

### Cytoplasmic p21 is more abundant in resistant cells than in sensitive cells

**Figure 2:**
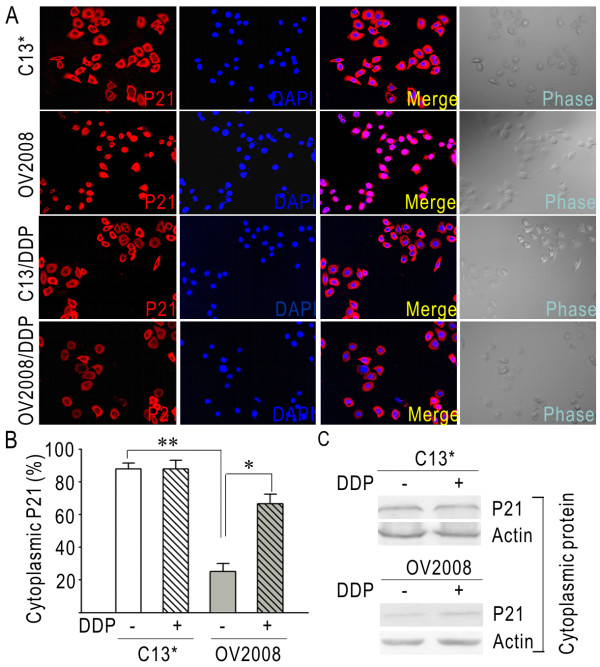
Cellular distribution of p21 in C13* and OV2008 cells and its alteration after 3-week of continuous cisplatin treatment. (A) Localization of p21 in C13* (upper panels), OV2008 (upper middle panels), C13*/DDP (lower middle panels) and OV2008/DDP (lower panels) cells. (B) Percentage of cytoplasmic p21 positive cells. (C) Comparison of cytoplasmic p21 between C13* and C13*/DDP, and between OV2008 and OV2008/DDP. (*, p < 0.05; **, p < 0.01).

Expression of p21 was examined by real-time PCR and western blot in the paired cell lines. There was no significant difference in the levels of p21 mRNA and total protein between the two cell lines (Figure 1C and 1D). The result of real-time PCR was normalized to the expression of GAPDH. The density of individual bands from western blot results were quantified and the ratios of C13* to OV2008 were calculated. The ratios of C13* to OV2008 in real-time PCR and western blot were 0.99 and 1.05, suggesting that C13* and OV2008 cells had equal expression levels of p21 (Figure 1D) (p > 0.05). By comparing cytoplasmic protein levels, we found that the amount of cytoplasmic p21 was much higher in C13* cells than in OV2008 cells (Figure 1C). The relative densities of the western blot bands shown in Figure 1C were calculated and the value was shown in Figure 1D. The results demonstrate that C13* cells have a 3.31 (± 0.33)-fold higher level of cytoplasmic p21 than do OV2008 cells (p < 0.05). We used immunofluorescence and confocal microscopy to further validate these differences and found that in C13* cells p21 was predominantly located in cytoplasm (Figure 2A, upper panels), while in OV2008 cells p21 was mainly restricted in the nucleus (Figure 2A, upper middle panels). In addition, we determined the percentage of cytoplasmic p21 in C13* and OV2008 cells. As shown in Figure 2B, we found that in C13* cells 88.1% ± 3.45% of p21 was found in the cytoplasm, while in OV2008 cells 25.2% ± 4.8% of p21 was cytoplasmic (p < 0.05).

### Persistent exposure to low dose cisplatin induces p21 translocation into cytoplasm in sensitive cells

To examine the effect of cisplatin on p21, OV2008 and C13* cells were consecutively treated with low-dose of 5 μM cisplatin. Three weeks later, immunofluorescence and confocal microscopy were applied to detect p21's expression and distribution. In OV2008 cells treated with cisplatin 66.6% ± 5.96% of p21 was found in the cytoplasm (Figure 2A, lower panels), which was dramatically higher than in OV2008 cells not treated with cisplatin (Figure 2B) (p < 0.05). However, in C13* cells no obvious changes of p21 sub-cellular location was observed after treatment with cisplatin (Figure 2A, lower middle panels). The percentage of cytoplasmic p21 in C13* cells that were exposed to cisplatin was 87.9% ± 5.22%, which was equal to parental control (Figure 2B) (p > 0.05). By measurement of western blot, the amount of cytoplasmic p21 was notably enhanced after treating with cisplatin in OV2008 (Figure 2C). However, there was no significant elevation in cytoplasmic p21 protein in C13* cells after treating with cisplatin (Figure 2C).

### Knockdown of cytoplasmic p21 restores the sensitivity to cisplatin in C13*

**Figure 3:**
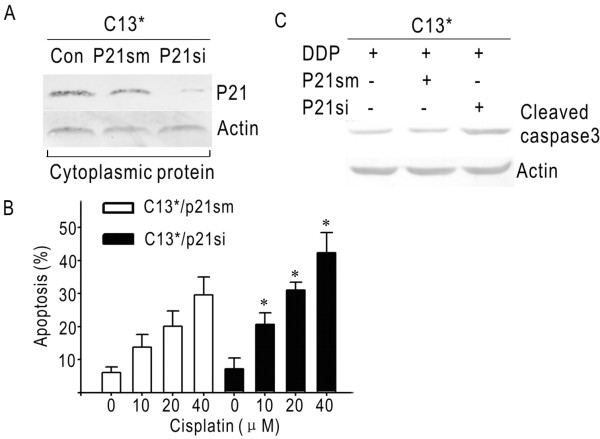
**Knockdown of cytoplasmic p21 in cisplatin-resistant cells enhances apoptosis rate to cisplatin**. (A) Representative western blot demonstrating the changes in cytoplasmic p21 protein levels observed in C13*/control, C13*/p21si and C13*/p21sm cells. (B) Apoptosis rates in response to cisplatin. (C) Corresponding western blot images of each group of cells depicted in A showing changes in cleaved caspase 3. (*, p < 0.05).

To determine whether cytoplasmic p21 contributes to cisplatin resistance, RNA interference assay in C13* was applied to suppress p21 that was mainly in the cytoplasm. C13* cells were transiently transfected with p21si and its mismatched fragment of p21sm. By western blot, p21si transfection exhibited a dramatic decrease in cytoplasmic p21 compared with control groups (Figure 3A). After transfection, these cells were exposed to different concentrations of cisplatin for 48 hours. As shown in Figure 3B, the apoptosis rates in both C13*/p21sm and C13*/p21si exhibited a concentration-dependent manner. The rates in p21si group were notably higher than their counterparts in mismatch-transfected group (p < 0.05). In the meantime, the effect of cytoplasmic p21 on cisplatin sensitivity was examined by caspase 3 activation. Using western blot, we found the level of cleaved caspase 3 protein in p21si group was significantly higher than its control groups (Figure 3C). These data indicate the reduction of cytoplasmic p21 endows cisplatin-resistant cells with increased sensitivity through activation of caspase 3.

### Inhibition of p21 translocation into cytoplasm enhances the sensitivity to cisplatin in C13*

**Figure 4:**
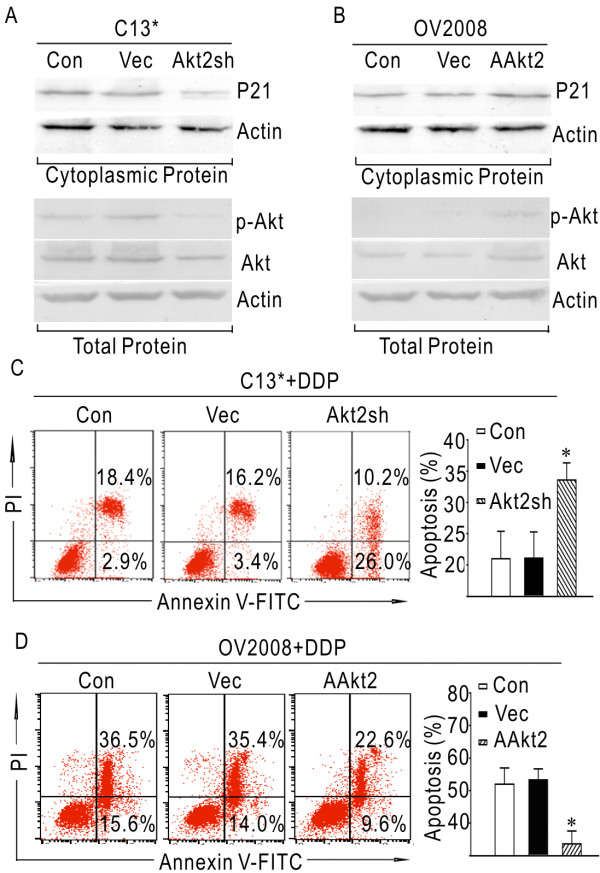
Inhibition of p21 translocation into cytoplasm enhances the sensitivity to cisplatin in C13*, while induction of p21 translocation into the cytoplasm results in increased resistance to cisplatin in OV2008. (A) Representative western blot images showing the changes in Akt, p-Akt and cytoplasmic p21 in C13*. (B) Representative western blot images showing the changes in Akt, p-Akt and cytoplasmic p21 in OV2008. (C) Apoptosis rates of C13* cells in response to cisplatin. (D) Apoptosis rates of OV2008 cells in response to cisplatin. (*, p < 0.05).

Expression of Akt and p-Akt was examined by western blot in the paired cell lines. As shown in additional file 1, Figure S1, the protein level of p-Akt in C13* was markedly higher than in OV2008, while there was no significant difference in the Akt protein between the two cell lines. Short hairpin RNA targeting Akt2 was stably transfected into C13* cells. Total and cytoplasmic protein was extracted and analyzed by western blot. In contrast to control cells, the protein levels of Akt and p-Akt were significantly decreased in Akt2Sh transfected cells. In addition, cytoplasmic p21 was remarkably decreased following RNA silencing of Akt2 (Figure 4A). Flow cytometric analysis of cells exposed to 20 μM cisplatin for 48 hours demonstrated that C13* cells transfected with Akt2 exhibited 33.6% ± 2.7% apoptosis rate, which was higher than non-transfected control cells (21% ± 4.4%) and vector-transfected cells (21.1% ± 4.2%) (p < 0.05, Figure 4C). Collectively, these results demonstrate that inhibition of cytoplasmic p21 through inactivation of Akt2 restores sensitivity of resistant ovarian cells to cisplatin.

### Induction of p21 translocation into the cytoplasm decreases sensitivity to cisplatin in OV2008

The plasmid of AAkt2 was stably transfected into OV2008 cells. Total and cytoplasmic protein was extracted from the cells and analyzed by western blot. The protein levels of Akt and p-Akt were significantly increased in AAkt2 cells when compared to control cells (Figure 4B). Moreover, cytoplasmic p21 protein levels were markedly enhanced in AAkt2 transfected cells when compared to control cells (Figure 4B). Flow cytometric analysis of cells exposed to 20 μM cisplatin for 48 hours demonstrated that OV2008 cells transfected with AAkt2 exhibited lower levels of apoptosis (33.6% ± 4.0%) than did non-transfected control cells (52% ± 5.1%) and vector-transfected cells (53.3% ± 3.4%) (p < 0.05, Figure 4D). These results show that accumulation of p21 in cytoplasm by activation of Akt2 impairs the susceptibility of sensitive ovarian cells to cisplatin.

### Expression of p21 in the cytoplasm is associated with cisplatin response in clinical samples

**Figure 5:**
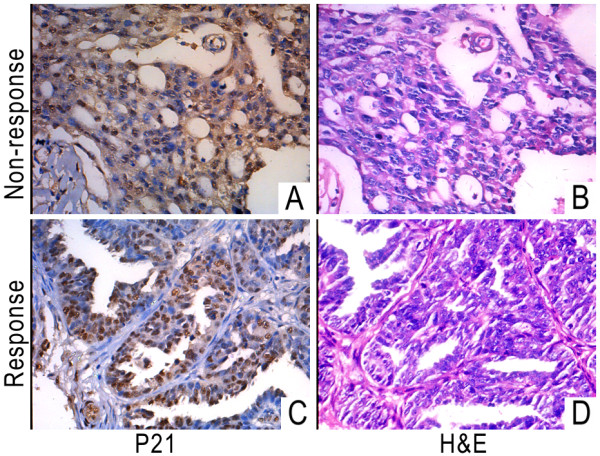
**Representative immunohistochemical staining for p21 and H&E analysis of tissues from treatment non-response (upper panels) and treatment response (lower panels) patients**. (A) Representative image of p21 expression in tissues from a treatment non-response patient. (B) H&E staining of a sequential section from the sample shown in A. (C) Representative image of p21 expression in tissues from a treatment response patient. (D) H&E staining of a sequential section from the sample shown in C.

Based on the results of our in vitro experiments, we decided to examine whether cytoplasmic p21 expression can serve as a marker for cisplatin resistance in ovarian cancer patients. Histological sections of ovarian cancer tissues were examined by immunohistochemistry for p21. The patients' clinical characteristics and results of p21 immunohistochemistry were shown in Table 1. The number of patients with cytoplasmic and non-cytoplasmic p21 staining was 5 and 8 in the non-response group. However, in the response group, 2 and 25 patients were cytoplasmic and non-cytoplasmic respectively. These findings indicated that the proportion of cytoplasmic p21 staining was significantly higher in non-response group than in response group (p = 0.027). Representative images of immunohistochemistry were shown in Figure 5, which demonstrated that p21 was predominantly localized in the cytoplasm in the non-response patient group (Figure 5A). In contrast, p21 is mainly localized in the nucleus in response group (Figure 5C). Additionally, cytoplasmic p21 staining was evaluated in patients at different clinical stages. In patients at stages III & IV, the number of patients with cytoplasmic and non-cytoplasmic p21 staining was 5 and 20, respectively. In patients at stages I & II, 2 patients exhibited cytoplasmic p21 staining, and 13 patients exhibited noncytoplasmic p21 staining (p = 0.691). This result suggested that there was no significant difference in the percentage of patients with cytoplasmic p21 staining between high stage and low stage ovarian tumors. Similarly, there was no significant difference in the levels of cytoplasmic p21 staining between serous and non-serous tumors (p = 0.407) or between groups of different ages (p = 0.677).

**Table 1:** Association of cytoplasmic p21 with clinicopathological parameters (n = 40)

Characteristics		Total	p21's expression		*P*#
				
			Cytoplasmic	Nuclear/Negative	
Age	≤ 50	23	5	16/2	.677
	> 50	17	2	14/1	
Histology*	Serous	20	2	17/1	.407
	Mucous	8	1	6/1	
	Others	8	3	5/0	
Stage	I & II	15	2	13/0	.691
	III & IV	25	5	17/3	
Cisplatin	Response	27	2	23/2	.027
	Non-response	13	5	7/1	

* Four patients are not available; *P *values are calculated by Fisher exact test.

## Discussion

It is well known that p21^(Waf1/Cip1) ^in the nucleus functions as a tumor suppressor by binding to cyclin/CDK complexes and proliferating cell nuclear antigen [15,16]. However, recent studies [31,32] have revealed that p21 can be a paradoxical tumor promoting factor and has been associated with poor cancer prognosis due to its accumulation in the cytoplasm. Recently, Koster has reported cytoplasmic p21 expression levels determined cisplatin resistance in testicular cancer [37]. This study was aimed at exploring the relationship between cytoplasmic p21 and cisplatin resistance in ovarian cancer, and investigating whether regulation of cytoplasmic p21 could alter the response to the therapy.

The cisplatin-resistant cell line C13* used in this study was established after in vitro challenge of OV2008 cells, a line derived from one ovarian carcinoma patient without prior chemotherapy, with cisplatin [38,39]. Since C13* and OV2008 cells shared the same genetic background and had minimum variation, they were superior to other cell lines and were chosen as optimal models for in vitro investigation of drug resistance. After confirming the expression levels and cellular distribution of p21 in the paired cell lines, we sought to determine whether it was influenced by cisplatin treatment and whether it was involved in cisplatin resistance. Long-term exposure to low-dose cisplatin resulted in p21 cytoplasmic translocation in cisplatin-sensitive cells, while the same treatment nearly had no effect on p21 cellular distribution in cisplatin-resistant cells. These results suggested p21's accumulation in cytoplasm was probably a protective response of sensitive cells to the drug, which helped them to escape from being killed by chemotherapeutics. Given that the endogenous p21 protein in resistant cells was mainly located in the cytoplasm, loss-of-function assay of cytoplasmic p21 was directly taken by RNA interference technology. Our results indicated knockdown of cytoplasmic p21 through p21 siRNA notably enhanced drug response in C13* cells. However, p21 transfection might not be the suitable strategy to illustrate the influence of increased cytocymic p21 because endogenous p21 in OV2008 was mainly restricted in the nuclear; The exogenous p21 transfected into OV2008 would probably mainly increase the nuclear amount instead of cytoplasmic amount. Thus, we increased the amount of cytoplasmic p21 by promoting its translocation from nuclear to cytoplasm in sensitive cells.

Zhou et al. reported that activation of phosphatidylinositol 3-kinase (PI3K)/Akt signaling can stimulate cytoplasmic accumulation of p21 [29]. There are three isoforms of Akt: Akt1, Akt2 and Akt3. All three isoforms share a high degree of amino acid sequence identity, especially within the kinase domain [40], and are activated by similar pathways in a PI3K-dependent manner. Overexpression of Akt2 has been found in 20% of human ovarian cancers [41], and increased Akt2 kinase activity has been found in approximately 30% of ovarian cancers [42]. Given these findings, we performed experiments where we altered the levels of cytoplasmic p21 by modulating Akt2 signaling. In OV2008 cells, a constitutively active AAkt2 plasmid was applied to activate PI3K/Akt signaling. Our results showed that the AAkt2 plasmid efficiently activated phosphorylation of Akt and, more importantly, significantly promoted the translocation of p21 into the cytoplasm. In the meantime, functional inhibition of cytoplasmic p21 was accomplished by short hairpin RNA silencing of Akt2 in C13* cells. Nevertheless, in fibroblasts and myoblasts, it has been suggested that the accumulation of p21 in the cytoplasm is stimulated by Akt1 [43]. The differences in our results might be ascribed to the different cell types used in the studies, similarity among three Akt isoforms and the mutual activation of the different isoforms. Therefore, the role of different Akt isoforms in the cytoplasmic translocation of p21 requires further investigation.

Caspase 3 activation is considered to be a key cellular component of the terminal and irreversible phase of apoptotic death caused by DNA damaging agents. In our experiments using flow cytometry, we found that the levels of cleaved caspase 3 (17 kDa) were proportional to the rate of apoptosis. Previously, it was reported that p21 could bind to procaspase 3 [31] and prevent its conversion to mature caspase 3, leading to the inhibition of apoptosis. In our experiments, it is likely that caspase 3 activation is inhibited by cytoplasmic p21; however, further studies are required to confirm this hypothesis.

By immunohistochemical analysis we found that a large proportion of tissues from treatment non-response tumors stained positive for cytoplasmic p21 (5 to 8) when compared to tissues from the treatment response group (2 to 25) (p = 0.027). We also showed that there was no significant difference in the level of cytoplasmic p21 staining between high stage and low stage ovarian tumors (p = 0.691), and between patients of different ages (p = 0.677). Furthermore, we compared cytoplasmic p21 levels in serous and non-serous tumors because the number of mucous and other types of tumors was limited. However, information on the prognosis and survival of the patients was not available; therefore, our investigation could not make a statistical analysis between cytoplasmic p21 and disease-free or survival rates.

## Conclusions

Our in vitro experiments demonstrate cytoplasmic p21 is a novel biomarker in cisplatin resistance, and regulation of cytoplasmic p21 could alter the response of ovarian tumor cell lines to the drug. This finding supplements our previous studies [11,12,34] and verifies that p21 is a downstream effector in the PI3K/Akt2 pathway that contributes to drug resistance.

## Abbreviations

siRNA: small interference RNA; shRNA: short hairpin RNA; CDK: cyclin-dependent kinase; PI3K: phosphatidylinositol 3-kinase; PTEN: phosphatase and tensin homolog; XIAP: X-linked inhibitor of apoptosis; MKP3: mitogen-activated protein kinase phosphatase 3; MDR: multidrug resistance protein.

## Competing interests

The authors declare that they have no competing interests.

## Authors' contributions

XX and QM carried out the immunofluorescence staining, participated in functional assay and drafted the manuscript. XL, TJ, PC and HX participated in nucleus p21 siRNA sequence designing and silencing efficiency. KL, YF and DW performed cell viability assay, western blot and RT-PCR. YW, SL and ZH collected clinical data, and carried out flow cytometry and immunohistochemistry assay. RL and TZ performed statistical analysis. GX participated in the data interpretation and provided expertise in molecular biological techniques. SW was responsible for writing and revising the manuscript. JZ, DM and LM participated in the design and coordination of the study. All authors have read and approved the final manuscript.

## Pre-publication history

The pre-publication history for this paper can be accessed here:

http://www.biomedcentral.com/1471-2407/11/399/prepub

## Supplementary Material

Additional file 1 **Figure S1**. Representative western blot depicting the Akt and p-Akt protein levels between C13* and OV2008 cells. Total protein was extracted from C13* and OV2008, and western blot was applied to compare the Akt and p-Akt expression between the paired cell lines. Click here for file
