# Supplementary figures and images for: Cytoplasmic p21 is a potential predictor for cisplatin sensitivity in ovarian cancer
Source: BMC Cancer. 2011 Sep 21;11:399. doi: 10.1186/1471-2407-11-399 (PMC3184122; doi:10.1186/1471-2407-11-399)

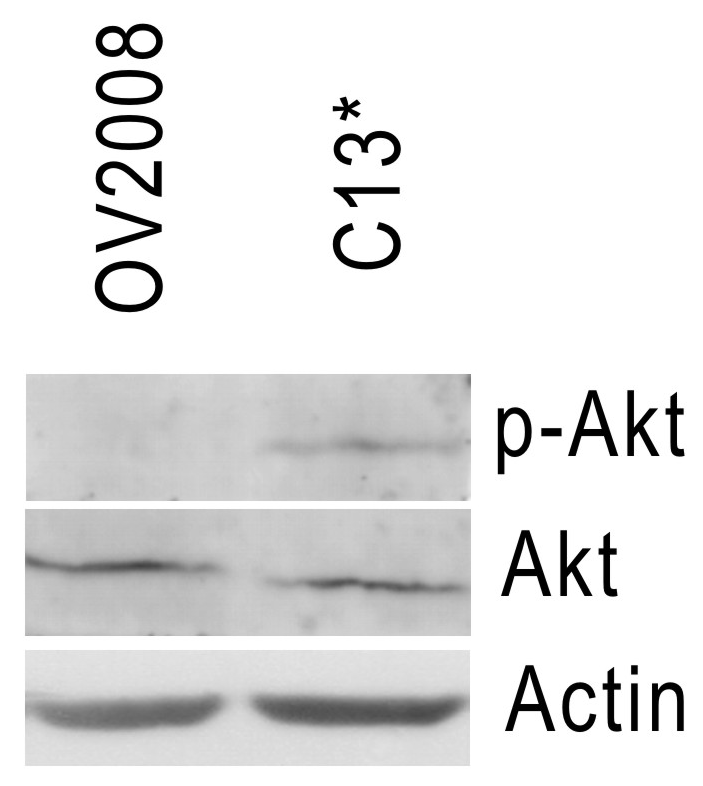

Supplement: Additional file 1 — Figure S1. Representative western blot depicting the Akt and p-Akt protein levels between C13* and OV2008 cells. Total protein was extracted from C13* and OV2008, and western blot was applied to compare the Akt and p-Akt expression between the paired cell lines. [file 1471-2407-11-399-S1.TIFF]
